# In Hamstring Muscles of Patients With Knee Osteoarthritis an Increased Ultrasound Shear Modulus Indicates a Permanently Elevated Muscle Tonus

**DOI:** 10.3389/fphys.2021.752455

**Published:** 2022-01-25

**Authors:** Feng Li, Zhen-Ya Wang, Zhi-Jie Zhang, Su-Hong Shen, Jia-Yi Guo, Yan-Xing Guo, Yi-Ran Feng, Lin Zhang, Yong-Bing Wen, Yun-Fei Zhang, Yi-Ming Fan, Meng-Meng Fan

**Affiliations:** ^1^Luoyang Orthopedic Hospital of Henan Province (Orthopedic Hospital of Henan Province), Luoyang, China; ^2^New Zealand College of Chinese Medicine, Auckland, New Zealand

**Keywords:** elasticity imaging techniques, hamstring, shear modulus, ultrasonography, osteoarthritis

## Abstract

**Background:**

Some patients with knee osteoarthritis (KOA) show pain, stiffness and limited flexion and extension at the back of the knee, leading to dysfunction and affecting life. This may be related to changes in the biomechanical properties of skeletal muscles. Shear wave elastography (SWE) can detect these changes by measuring muscle shear modulus.

**Aims:**

To investigate hamstring muscle shear modulus of healthy people and patients was studied using SWE method, and the correlation analysis between the Western Ontario and McMaster Universities Osteoarthritis Index (WOMAC) score of patients’ subjective feeling and shear modulus of objective quantification was conducted.

**Methods:**

The hamstring shear modulus was measured by SWE in 50 patients and 50 healthy individuals. Pearson correlation coefficient was used to evaluate the correlation between hamstring stiffness and shear modulus in patients.

**Results:**

The hamstring shear modulus were significantly higher in the KOA group [the semimembranosus (SM) 15.23 ± 7.23, the semitendinosus (ST) 15.94 ± 5.40, the biceps femoris long tendinitis (BFL) 14.21 ± 6.55] than in the control group (the SM 10.95 ± 2.41, the ST 11.25 ± 2.23, the BFL 9.98 ± 2.81) (*p* = 0.000, *p* = 0.000, *p* = 0.001). The hamstring shear modulus in the KOA group was moderately positively correlated with pain, shear modulus, and physical function score.

**Conclusion:**

Preliminary results show that the shear modulus of the hamstring of KOA patients is higher than that of healthy people, the WOMAC score and the shear modulus of patients are moderately correlated. These preliminary results show that ultrasonic shear wave elastography measurement of shear modulus may be enough to sensitive, can detect these effects, more targeted in order to assist the doctor’s diagnosis and treatment.

## Introduction

Knee osteoarthritis (KOA) is the most common form of arthritis, and the leading cause of disability and pain affecting middle-aged and elderly people worldwide (Reginster, 2002; Kurtais et al., 2011), caused by structural changes in joints resulting in pain, deterioration of function, and disability (Ding et al., 2007; Hunter, 2007). In clinical practice, we found that some patients with KOA presented with stiffness in the back of the knee, pain in traction, limited flexion and extension, and even abnormal gait. It has been hypothesized that patients with KOA present with excessive muscular co-contraction during gait which may help stiffen and stabilize the knee during gait through increased compressive forces and joint loading (Lewek et al., 2004; Mills et al., 2013; Brandon et al., 2014). Skeletal muscle undergoes structural changes with abnormal prolonged contraction of muscles which may alter its biomechanical properties. Histologically, changes in biomechanical properties is associated with changes in muscle composition including myosteatosis, myofibrosis (Faulkner et al., 2007) and dysfunction in extracellular elastic fibers (Kragstrup et al., 2011). Such changes may alter the biomechanical properties resulting in changes to muscle stiffness, limited movement, muscle pulling pain. Therefore, it is meaningful to evaluate the biomechanical properties of the muscle around the knee to further explore its biomechanical mechanism and provide standard guidance in rehabilitation plans.

Large number of studies have shown that the increase in muscle tension in patients is accompanied by changes in muscle stiffness. Guo et al. (2016) found that patients with neck and shoulder myofascial pain have increased trapezius muscle tone, and SWE can detect that the shear modulus of the trapezius muscle is higher than that of normal people. Mitsuhiro Masaki, Tomoki Aoyama and other studies found that medical staff with low back pain were accompanied by low back muscle stiffness (Masaki et al., 2017). SWE found that the multifidus muscle elastic modulus of the low back pain group was higher than that of the asymptomatic group. They believed that the back pain was related to the stiffness of the multifidus muscle. In the clinic, we found that some KOA patients have symptoms of stiffness on the back of the knee, traction pain, and limited mobility (Michael et al., 2010; Sharma, 2021). We believe that these symptoms are related to the stiffness of the hamstrings. At present, the muscle stiffness on the back of the knee has not been clearly clarified because it is often difficult to quantitatively assess the stiffness of individual muscles. However, it has become possible to use ultrasonic shear elasticity imaging to measure the shear elastic modulus. As early as 1991, the technology of ultrasound elastography was first proposed by Ophir et al. (1991). The basic principle is to display the different hardness of the tissue according to the degree of deformation of the tissue after being stressed, thereby reflecting the occurrence and development of the disease, and providing help for clinical diagnosis and treatment. Subsequently, in 1998, the shear wave elastic imaging technology proposed by Sarvazyan et al. (1998) and Catheline et al. (1999). The principle is that the sound source vibrates to generate sound waves. The tissue particles are caused to vibrate laterally, thereby generating shear waves, and by tracking the propagation speed of the shear waves, the absolute value of the tissue elasticity-Young’s modulus (*E* = 3ρct^2^, E: Young’s modulus; c: shear wave propagation velocity; ρ: tissue density) is obtained, so as to quantitatively analyze and compare the elastic differences between the organizations, aid in clinical diagnosis.

Shear modulus of elasticity is used as an indicator of muscle stiffness. Previous studies have shown that SWE is an appropriate and reliable method for evaluating the shear modulus of muscle (Leong et al., 2013; MacDonald et al., 2016). The purpose of this study was to explore the difference in hamstring shear modulus between KOA patients and normal people, and the relationship between WOMAC subjective function score and hamstring shear modulus in KOA patients.

The Western Ontario and McMaster Universities Osteoarthritis Index (WOMAC) score is widely used and has been validated mainly in the context of KOA since the 1990s (Bellamy et al., 1988; Davies et al., 1999; Escobar et al., 2002). It evaluates pain, function, and stiffness during everyday activities. Each score ranges from 0 to 10, with 10 being the worst outcome. But WOMAC functional scoring is based on the subjective feelings of the patient, objective quantitative analysis of muscle in patients with KOA is still lacking. Ultrasound shear wave elastography (SWE) is a new quantitative method used for assessing tissue shear modulus. It has been widely utilized in breast, liver and thyroid, it is also gradually applied to the research of skeletal muscle system (Sigrist et al., 2017). SWE can be used as a non-invasive diagnostic tool to evaluate the elastic modulus of soft tissues such as muscle ligaments in real time. It is simple to operate and has no radiation hazards. At the same time, it not only used to observe the overall shape of the soft tissue, but also to set interesting areas of the soft tissue to be inspected. Through the color depth, we can directly show the small elasticity of the target tissue. Moreover, through SWE, the elastic modulus of the soft tissue in the loosening state, active motion, passive motion state, and physical state and pathological state can be accurately evaluated.

In clinical practice, we found that some patients with KOA have symptoms of posterior knee stiffness, traction pain, and limited flexion and extension. We believe these symptoms are related to the shear modulus of the hamstring. Therefore our primary aim was to investigate to what extent hamstrings muscle shear modulus measured by SWE differed amongst KOA patients and healthy people of the same age. The secondary aim was to investigate the correlation between hamstring muscle shear modulus measured by SWE and WOMAC scores of subjective feelings in KOA patients.

## Materials and Methods

### Ethical Approval

The study was approved by the Ethics Committee of Luoyang Orthopedic Hospital in Henan Province. Prior to the study, the steps of the experiment were fully explained to each subject. All subjects provided written informed consent before the experiment, and all research procedures followed the principles of the Helsinki Declaration.

### Subject

We recruited KOA patients aged 50–60 years, all of whom had symptoms of posterior knee stiffness, posterior knee traction pain, and limited flexion and extension. Among them, there were 22 males and 28 females (age 54.68 ± 3.25 years old), an average height of 163.46 cm and an average weight of 69.35 kg. Meanwhile, we recruited healthy subjects aged between 50 and 60, including 18 males and 32 females (age 53.85 ± 2.16 years old), their average height was 163.50 cm, and their average weight was 70.61 kg. At the same time, subjects were selected based on the following factors (Alfuraih et al., 2019): (1) no previous history of musculoskeletal or neurological disorders; (2) not currently taking or previously taken a corticosteroid treatment for the past 3 years with doses >5 mg/day; (3) not currently taking or previously taken a HMG-CoA reductase inhibitors (statins) for the past 3 years. The Luoyang Orthopedic Traumatological Hospital of Henan Province (Henan Provincial Orthopedic Hospital) approved this study, and each subject provided written informed consent to participate.

### Ultrasound Imaging With Shear-Wave Elastography

Shear wave elastography was performed using the two-dimensional Aixplorer (Supersonic Imagine, Aix-en-Provence, France) system using the SuperLinear SL10-2 MHz probe. The scan was limited to the dominant side since limb dominance had no significant impact on muscle shear modulus (Alfuraih et al., 2018). Before scanning, all participants were placed in a prone position on the scanning bed and were asked to relax and be comfortable for 5 min. All participants were asked to refrain from any strenuous or sporting activities at least 1 day prior to the study to minimize possible confounding exercise effect. The transducer was oriented in the transverse plane over the region of interest on the measured muscle, and then the mode of SWE was activated to examine the shear wave modulus of the muscle or tendon. During the acquisition of the mode of SWE, the transducer was kept motionless for above 3 s (Zhou et al., 2019). Then the gray scale image was shown the appearance of the muscle under the longitudinal section. Image quality was closely monitored throughout measures. When the color in the ROI was uniform and several muscle fibers were continuously visible, the images were frozen, and then put the Q-box to obtain the shear wave modulus from the system and stored for off-line analysis (kPa) (Zhou et al., 2020). Three images were captured at each measurement site of tendon and muscle. The mean of the shear modulus from all three images were used for further analyses.

The hamstrings measurement position: (Alfuraih et al., 2017, 2019).

(1) Semimembranosus (SM): the junction of lower 1/3 and middle 1/3 of the mid-point of the popliteal transverse stripes from the mid-point of the gluteal transverse stripes. (2) Semitendinosus (ST): posterior medial side of the thigh, across the gluteal line to the middle of the popliteal line. (3) Biceps femoris long tendinitis (BFL): posterolateral thigh parallel to semitendinosus.

From each elastography image, the spatial average of the shear modulus (kPa) in an 8-mm circular area was determined. The musculoskeletal mode was used to estimate the shear modulus of the upper trapezius muscle with temporal averaging (persistence), penetration mode, and 85% opacity. The range of the color scale was adjusted from 0 to 200 kPa. The values were averaged across three measurements in each condition. The probe was placed on top of the skin with a minimal load ensuring no external pressure could affect the measurements.

### Western Ontario and McMaster Universities Osteoarthritis Index Knee Osteoarthritis Evaluation Scale

The WOMAC has demonstrated reliability, validity and responsiveness in patients with KOA (Bellamy et al., 1988). It is comprised of sub-scales (24 items), including pain (5 items), stiffness (2 items), and physical function (17 items). This scale, which evaluated osteoarthritis-related disability in the hip and/or knee osteoarthritis, is also very sensitive to the changes in health states. As scores increase in all dimensions, the intensity and severity of the complaint also increase (Bellamy, 2002).

### Data Analysis

Data analysis was performed using the SPSS 22.0 program. The cases distribution was evaluated by Kolmogorov–Smirnov test, and according to the results of this test, there was normal distribution. For that reason, we have used parametric tests for statistical evaluations to compare means. Pearson correlation analysis was used to evaluate the correlation between the hamstring shear modulus and WOMAC score. *p* values of less than 0.05 were defined as statistically significant.

## Results

### Demographic Data

Demographic information including age, gender, and BMI, for all subjects are shown in Table 1. Forty-six patients were included in the KOA group. Forty-six healthy subjects served as control group. These two groups were similar regarding demographic findings (Table 1).

**TABLE 1:** Patients’ demographic findings.

		Observation (*N* = 50)	Control (*N* = 50)	*p* value
Age (years)		56.87 ± 8.41	56.15 ± 8.94	0.693
Gender	Male	22 (44%)	18 (36%)	0.414
	Female	28 (56%)	32 (64%)	
BMI (kg/m^2^)		24.60 ± 3.67	23.6 ± 3.43	0.872

*BMI, body mass index. Data are expressed as frequency (percentage) or mean ± standard deviation.*

### Intra- and Interoperator Reliabilities

The related statistical parameters for intra- and interoperator reliabilities for assessing the hamstrings shear modulus are summarized in Table 2. The mean shear modulus values of the SM, SL, and BFL were 12.91 kPa, 12.83 kPa, and 11.27 kPa for operator A in test 1; 12.37 kPa, 12.45 kPa, and 11.56 kPa for operator A in test 2; 12.32 kPa, 12.85 kPa, and 11.51 kPa for operator B. The intraclass correlation coefficient (ICC) values of intraoperator reliability were good in SM [ICC = 0.85; 95% Confidence Interval (CI) = 0.45–0.94; standard error measurement (SEM) <0.63 kPa; and MDC < 1.75 kPa], in SL (ICC = 0.84; 95% CI = 0.43–0.94; SEM < 0.62 kPa; and MDC < 1.72 kPa) and excellent in BFL (ICC = 0.94; 95% CI = 0.73–0.98 SEM < 0.86 kPa; and MDC < 2.38 kPa). The ICC values of interoperator reliability were excellent in SM (ICC = 0.97; 95% CI = 0.86–0.99; SEM < 0.63 kPa; and MDC < 1.75 kPa), in SL (ICC = 0.98; 95% CI = 0.88–0.99; SEM < 0.62 kPa; and MDC < 1.72 kPa) and in BFL (ICC = 0.98; 95% CI = 0.89–0.99; SEM < 0.86 kPa and MDC < 2.38 kPa).

**TABLE 2:** Intra- and interoperator reliabilities for assessing the three hamstrings stiffness.

	SM			SL			BFL		
	Mean ± SD	SEM	MDC	Mean ± SD	SEM	MDC	Mean ± SD	SEM	MDC
Operator A in test 1	12.91 ± 2.78	0.62	1.72	12.83 ± 2.76	0.62	1.72	11.27 ± 3.21	0.62	2.0
Operator A in test 2	12.37 ± 2.82	0.63	1.75	12.45 ± 2.23	0.50	1.39	11.56 ± 3.84	0.86	2.38
Operator B	12.32 ± 2.42	0.54	1.50	12.85 ± 2.64	0.59	1.64	11.51 ± 3.38	0.76	2.11
ICC*^a^* (95% CI)	0.84 (0.43–0.94)			0.85 (0.45–0.94)			0.94 (0.73–0.98)		
ICC*^b^* (95% CI)	0.97 (0.86–0.99)			0.98 (0.88–0.99)			0.98 (0.89–0.99)		

*ICC, intraclass correlation coefficient; CI, confidence interval; SEM (kPa), standard error of measurement of kPa; MDC (kPa), minimal detectable change; SD (kPa), standard deviation of kPa; kPa, kilo Pascal.*

*^a^Intraoperator reliability.*

*^b^Interoperator reliability.*

### Differences of the Hamstring Shear Modulus Between the Knee Osteoarthritis and Control

The comparison of the hamstring shear modulus between the two groups is demonstrated in Table 3. The hamstring shear modulus were significantly higher in the KOA group than in the control group (*p* = 0.000, *p* = 0.000, *p* = 0.001).

**TABLE 3:** Shear modulus (kPa) of the three hamstring in two groups.

	SM	ST	BFL
Observation (*N* = 50)	15.23 ± 7.23	15.94 ± 5.40	14.21 ± 6.55
Control (*N* = 50)	10.95 ± 2.41	11.25 ± 2.23	9.98 ± 2.81
*p* value	0.000	0.000	0.001

### Correlation Between the Hamstring Shear Modulus and Western Ontario and McMaster Universities Osteoarthritis Index Score in the Knee Osteoarthritis Group

The correlation between the hamstring shear modulus and WOMAC score in the KOA group is demonstrated in Table 4. The hamstring shear modulus in the KOA group was moderately positively correlated with pain, stiffness, and physical function score.

**TABLE 4:** Correlation between the three hamstring shear modulus and WOMAC score in the observation group.

		SM	ST	BFL
Pain	*r*	0.477	0.519	0.631
	*P*	0.001	0.000	0.000
Stiffness	*r*	0.437	0.461	0.539
	*P*	0.002	0.001	0.000
Physical function	*r*	0.605	0.543	0.605
	*P*	0.000	0.000	0.000

## Discussion

In the present study, we found that SWE is a feasible tool for evaluating hamstring muscle shear modulus in patients with knee osteoarthritis, with good intra- and interoperator reliabilities. This study is the first to compare the hamstring muscle shear modulus of patients with knee osteoarthritis with that of healthy volunteers. The results show that the hamstring muscle shear modulus is significantly different between healthy volunteers and patients with knee osteoarthritis. The hamstring muscle shear modulus of patients has a negative correlation with the functional score.

Cortez et al. (2015) reported that the medial gastrosoleus and tibialis muscles had fair to good levels of intraobserver (ICC, 0.42–0.70) and interobserver (ICC, 0.69–0.73) reliability. Taş et al. (2017) reported that intraobserver reliability (ICC, 0.91–0.92) and interday reliability (ICC, 0.81–0.83) were excellent, and interobserver reliability (ICC, 0.71) was good of SWE in the patellar tendon, and the intraobserver reliability (ICC, 0.93–0.94), interday reliability (ICC, 0.81–0.91), and interobserver reliability (ICC, 0.95) were perfect in the rectus femoris. Koppenhaver et al. (2018) reported that overall reliability estimates were fair to excellent with ICCs ranging from 0.44 to 0.92 in low back musculature, and reliability was higher in the lumbar multifidus muscles than the erector spinae muscles, slightly higher during contraction than during rest, and substantially improved by using the mean of three measurements.

In the present study, intraoperator reliability were good for assessing the SM (ICC = 0.85) and the SL (ICC = 0.84), intraoperator reliability was excellent for assessing the BFL (ICC = 0.94), but the interoperator reliability were excellent for assessing the SM (ICC = 0.97), the SL (ICC = 0.98), and the BFL (ICC = 0.98) corresponding to the results of SEM and MDC. The findings from this study have indicated that the SWE is a credible instrument for evaluating the hamstrings shear modulus.

According to the new findings of the preliminary study, the shear modulus of SM, ST, and BFL in patients with KOA with pain of posterior knee traction and limited flexion and extension significantly increased compared with normal people, and the higher the shear modulus of the muscles in the hamstring, the higher the WOMAC (pain, stiffness, physical function) score of patients’ subjective feelings. The increase in the shear modulus of the patient’s hamstring muscle reflects the change in muscle biomechanical properties, that is, the deterioration of the elasticity of the muscle tissue, which in turn affects the functional activities of the knee joint of the patient, which explains the correlation between the two. These results suggest that shear wave elastography can quantitatively detect changes in hamstring shear modulus in KOA patients, in the form of shear modulus, which is closely related to patients’ functional activities. These preliminary findings provide a proof-of-concept that would be beneficial to support this technique as it would assess the shear modulus of the diseased muscles of the patient and validate the WOMAC score. Many researchers have found that the technique can assess the effectiveness of interventions such as dry needles and manual interventions in muscles.

In the past (Hoang et al., 2009), two-dimensional ultrasound has been used to obtain rich morphological parameters, such as muscle thickness, cross-sectional area, muscle fiber length, feather horn, etc. To some extent (Cartwright et al., 2013), ultrasonic technology has been used to evaluate neuromuscular, tendon, ligament and joint lesions and guide rehabilitation treatment, but two-dimensional ultrasound cannot provide bone images. Mechanical properties of muscle system and anisotropy of muscle tissue also increase the limitations of two-dimensional ultrasound application (Botar et al., 2012). In recent years, French Aixplorer ShearWave real-time SWE ultrasonic diagnostic instrument was produced. Based on the automatic generation and analysis of real-time Shear Wave, young’s modulus value, as an important biomechanical parameter, was obtained through the quantitative analysis system, so as to realize the full quantification of ultrasonic elastography and promote the extensive clinical research of SWE.

Rodrigues and Rodrigues Junior (2000) proposed that the elastic fiber system in the muscle extracellular matrix gradually loses its resistance with age, promoting the loss of normal muscle shear modulus. According to Alfuraih et al. (2019) research on the muscles of the lower limbs, the shear modulus of resting muscles gradually decreases throughout the adult life, especially in the elderly (>75 years old). Obviously, these are studies of healthy people. Maher et al. (2013) study showed that the shear modulus of the trapezius muscle under the action of myofascial trigger points (MTrPs) was sensitive enough to detect the change of the dry needle treatment and the influence of posture on the shear modulus. The cause (Hubbard and Berkoff, 1993; Simons, 1996) may be abnormal activity in the endplate of neuronal movement at the end of muscle fibers at the MTrPs. Continuous muscle segment contracture leads to increased local metabolic demands, pressure on capillary circulation, and increased metabolic by-products, resulting in local muscle shear modulus. However, the etiology and pathophysiology associated with KOA are currently being studied but remain unclear. This study starts from the mechanical properties of muscle and discusses the relationship between muscle and function. To our knowledge, this is the first study to combine subjective functional scores with objective quantitative indicators.

**FIGURE 1:**
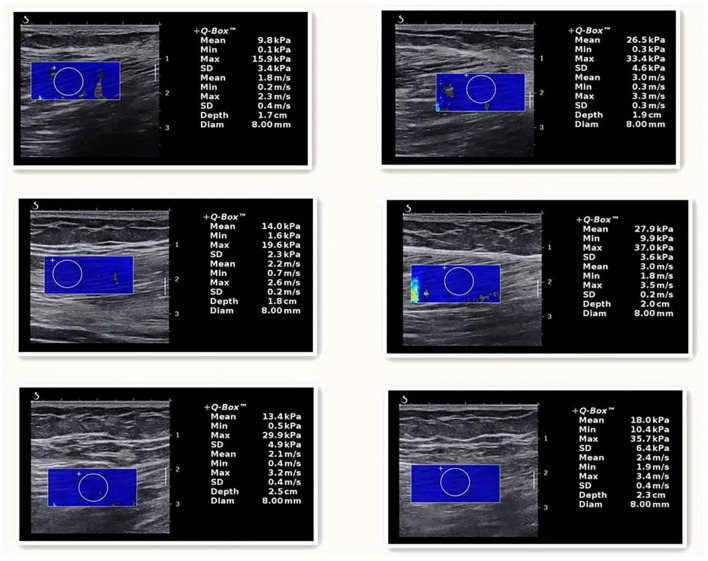
The one on the left is listed as a healthy person, and the SM, ST, and BFL are listed from top to bottom. The one on the right is a patient, and the SM, ST, and BFL are listed from top to bottom.

Our findings show that tissue shear modulus can be visualized in real time, while the difference between the sick and the healthy are readily observed and displayed via a color-coded map (Figure 1). These findings may shed more light on the mechanisms by which patients experience pain, stiffness, and limited functioning. This study demonstrated the feasibility of SWE techniques for assessing muscle stiffness in KOA patients. But the muscle assessed in this study was a superficial muscle that is easy to palpate. The same cannot be said for deeper muscles such as those in the lumbar region or deep abdominal area. Consequently, an inability to palpate and reproduce the classic referral patterns is difficult to accomplish for the clinician. Shear wave elastography would provide such a means for an objective evaluation of tissues of different types and depths, and a means to determine the efficacy and longevity of therapeutic interventions. Currently (Kwon et al., 2012; Berko et al., 2014), there are still no relevant reports on the normal young’s modulus of muscle tissue and the reference range of abnormal values, which will become the future research direction and play a greater role in the diagnosis of muscle injury, monitoring the development of diseases and evaluating the curative effect of diseases.

## Conclusion

**FIGURE 2:**
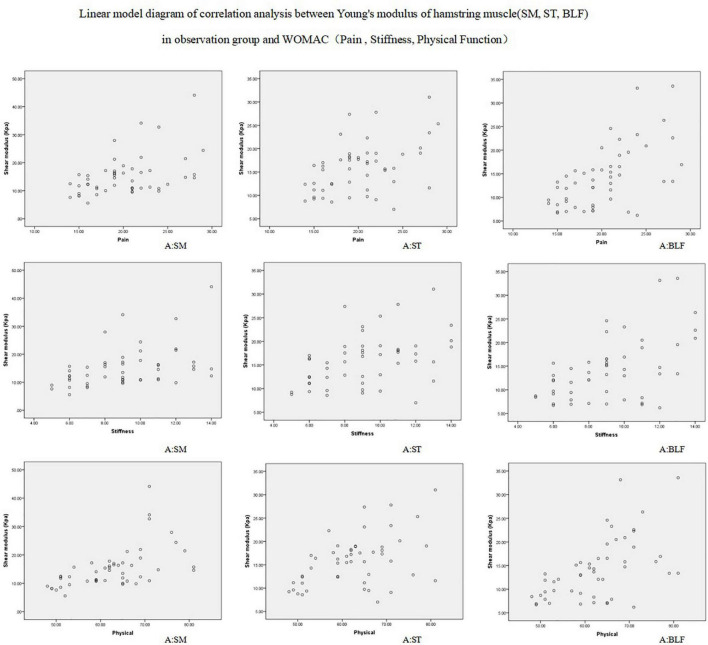
Correlation between the three hamstring shear modulus and WOMAC score in the observation group.

Preliminary results show that the hardness of KOA patients hamstring were higher than in healthy people, show that muscle hardness change is a major feature of KOA patients, the patients’ subjective aspect WOMAC score has a moderate correlation, the greater the hardness of muscle, feel stiff and dysfunction in patients with more serious (Figure 2), these preliminary results show that ultrasonic shear wave elastography measurement of shear modulus may be enough to sensitive, can detect these effects, more targeted in order to assist the doctor’s diagnosis and treatment.

## Data Availability Statement

The raw data supporting the conclusions of this article will be made available by the authors, without undue reservation.

## Ethics Statement

The study was approved by the Ethics Committee of Luoyang Orthopedic Traumatological Hospital of Henan Province. Prior to the study, the steps of the experiment were fully explained to each subject. All subjects provided written informed consent before the experiment, and all research procedures followed the principles of the Helsinki Declaration.

## Author Contributions

FL and Z-YW designed the study and drafted the manuscript. J-YG, Y-XG, and FL helped to conceive the study. Z-JZ, S-HS, and Y-RF helped to perform the statistical analysis. LZ, Y-BW, Y-FZ, Y-MF, and M-MF participated in the data collection. All authors reviewed and approved the final manuscript.

## Conflict of Interest

The authors declare that the research was conducted in the absence of any commercial or financial relationships that could be construed as a potential conflict of interest.

## Publisher’s Note

All claims expressed in this article are solely those of the authors and do not necessarily represent those of their affiliated organizations, or those of the publisher, the editors and the reviewers. Any product that may be evaluated in this article, or claim that may be made by its manufacturer, is not guaranteed or endorsed by the publisher.

## References

[B1] Alfuraih A. M. O’Connor P. Hensor E. Tan A. L. Emery P. Wakefield R. J. (2018). The effect of unit, depth, and probe load on the reliability of muscle shear wave elastography: variables affecting reliability of SWE. *J. Clin. Ultrasound* 46 108–115. 10.1002/jcu.22534 28990683

[B2] Alfuraih A. M. O’Connor P. Tan A. L. Hensor E. Emery P. Wakefield R. J. (2017). An investigation into the variability between different shear wave elastography systems in muscle. *Med. Ultrasonogr.* 19 392–400. 10.11152/mu-1113 29197916

[B3] Alfuraih A. M. Tan A. L. O’Connor P. Emery P. Wakefield R. J. (2019). The effect of ageing on shear wave elastography muscle stiffness in adults. *Aging Clin. Exp. Res.* 31 1755–1763. 10.1007/s40520-019-01139-0 30762201 PMC6825644

[B4] Bellamy N. (2002). *WOMAC Osteoarthritis Index User Guide.* Brisbane, Qld: Nicholas Bellamy.

[B5] Bellamy N. Buchanan W. W. Goldsmith C. H. Campbell J. Stitt L. W. (1988). Validation study of WOMAC: a health status instrument for measuring clinically important patient relevant outcomes to antirheumatic drug therapy in patients with osteoarthritis of the hip or knee. *J. Rheumatol.* 15 1833–1840. 3068365

[B6] Berko N. S. Fitzgerald E. F. Amaral T. D. Payares M. Levin T. L. (2014). Ultrasound elastography in children: establishing the normal range of muscle elasticity. *Pediatr. Radiol.* 44 158–163. 10.1007/s00247-013-2793-z 24104402

[B7] Botar J. C. Vasilescu D. Damian L. Dumitriu D. Ciurea A. Dudea S. M. (2012). Musculoskeletal sonoelastography. pictorial essay. *Med. Ultrason.* 14 239–245. 22957331

[B8] Brandon S. C. Miller R. H. Thelen D. G. Deluzio K. J. (2014). Selective lateral muscle activation in moderate medial knee osteoarthritis subjects does not unload medial knee condyle. *J. Biomech.* 47 1409–1415. 10.1016/j.jbiomech.2014.01.038 24581816

[B9] Cartwright M. S. Kwayisi G. Griffin L. P. Sarwal A. Walker F. O. Harris J. M. (2013). Quantitative neuromuscular ultrasound in the intensive care unit. *Muscle Nerve* 47 255–259. 10.1002/mus.23525 23041986

[B10] Catheline S. Wu F. Fink M. (1999). A solution to diffraction biases in sonoelasticity:the acoustic impulse technique. *J. Acoust. Soc. Am.* 105 2941–2950. 10.1121/1.426907 10335643

[B11] Cortez C. D. Hermitte L. Ramain A. Mesmann C. Lefort T. Pialat J. (2015). Ultrasound shear wave velocity in skeletal muscle: a reproducibility study. *Diagn. Interv. Imaging* 97 71–79. 10.1016/j.diii.2015.05.010 26119864

[B12] Davies G. M. Watson D. J. Bellamy N. (1999). Comparison of the responsiveness and relative effect size of the western ontario and mcmaster universities osteoarthritis index and the short-form medical outcomes study survey in a randomized, clinical trial of osteoarthritis patients. *Arthritis Care Res.* 12 172–179. 10.1002/1529-0131(199906)12:3<172::AID-ART4>3.0.CO;2-Y 10513507

[B13] Ding C. Cicuttini F. Jones G. (2007). Tibialsubchondral bone size and knee cartilagedefects: relevance to knee osteoarthritis. *Osteoarthritis Cartilage* 15 479–486. 10.1016/j.joca.2007.01.003 17291789

[B14] Escobar A. Quintana J. M. Bilbao A. Azkárate J. Güenaga J. I. (2002). Validation of the Spanish version of the WOMAC questionnaire for patients with hip or knee osteoarthritis. Western Ontario and McMaster Universities Osteoarthritis Index. *Clin. Rheumatol.* 21 466–471. 10.1007/s100670200117 12447629

[B15] Faulkner J. A. Larkin L. M. Claflin D. R. Brooks S. V. (2007). Age-related changes in the structure and function of skeletal muscles. *Clin. Exp. Pharmacol. Physiol.* 34 1091–1096. 10.1111/j.1440-1681.2007.04752.x 17880359

[B16] Guo L. Zhang C. Zhang D. Gao J. H. Liu G. H. Wang S. Q. (2016). Application of shear wave ultrasound elastography in cervical and shoulder myofascial pain syndrome. *China Orthoped. Traumatol.* 29 142–145. 27141783

[B17] Hoang P. Saboisky J. P. Gandevia S. C. Herbert R. D. (2009). Passive mechanical properties of gastrocnemius in people with multiple sclerosis. *Clin. Biomech. (Bristol, Avon)* 24 291–298. 10.1016/j.clinbiomech.2008.12.008 19185961

[B18] Hubbard D. R. Berkoff G. M. (1993). Myofascial trigger points show spontaneous needle EMG activity. *Spine* 18 1803–1807. 10.1097/00007632-199310000-00015 8235865

[B19] Hunter D. J. (2007). In the clinic. Osteoarthritis. *Ann. Intern. Med.* 147:Itc8-1-itc8-16. 10.7326/0003-4819-147-3-200708070-01008 17679702

[B20] Koppenhaver S. Kniss J. Lilley D. Oates M. Fernández-de-Las-Peñas C. Maher R. (2018). Reliability of ultrasound shear-wave elastography in assessing low back musculature elasticity in asymptomatic individuals. *J. Electromyogr. Kinesiol.* 39 49–57. 10.1016/j.jelekin.2018.01.010 29413453

[B21] Kragstrup T. W. Kjaer M. Mackey A. (2011). Structural, biochemical, cellular, and functional changes in skeletal muscle extracellular matrix with aging. *Scand. J. Med. Sci. Sports* 21 749–757. 10.1111/j.1600-0838.2011.01377.x 22092924

[B22] Kurtais Y. Oztuna D. Kutlay S. Hafiz M. Tennant A. (2011). Reliability, construct validity and measurementpotential of the ICF comprehensive coreset for osteoarthritis. *BMC MusculoskeletDisord.* 12:255. 10.1186/1471-2474-12-255 22067295 PMC3228679

[B23] Kwon D. R. Park G. Y. Lee S. U. Chung I. (2012). Spastic cerebral palsy in children: dynamic sonoelastographic findings of medial gastrocnemius. *Radiology* 263 794–801. 10.1148/radiol.12102478 22495685

[B24] Leong H. T. Ng G. Y. Leung V. Y. Fu S. N. (2013). Quantitative estimation of muscle shear elastic modulus of the upper trapezius with supersonic shear imaging during arm positioning. *PLoS One* 8:e67199. 10.1371/journal.pone.0067199 23825641 PMC3692441

[B25] Lewek M. D. Rudolph K. S. Snyder-Mackler L. (2004). Control of frontal plane knee laxity during gait in patients with medial compartment knee osteoarthritis. *Osteoarthr. Cartil.* 12 745–751. 10.1016/j.joca.2004.05.005 15325641 PMC3123521

[B26] MacDonald D. Wan A. McPhee M. Tucker K. Hug F. (2016). Reliability of abdominal muscle stiffness measured using elastography during trunk rehabilitation exercises. *Ultrasound Med. Biol.* 42 1018–1025. 10.1016/j.ultrasmedbio.2015.12.002 26746381

[B27] Maher R. M. Hayes D. M. Shinohara M. (2013). Quantification of dry needling and posture effects on myofascial trigger points using shear wave elastography. *Arch. Phys. Med. Rehabil.* 94 2146–2150. 10.1016/j.apmr.2013.04.021 23684553

[B28] Masaki M. Aoyama T. Murakami T. Yanase K. Ji X. Tateuchi H. (2017). Association of low back pain with muscle stiffness and muscle mass of the lumbar back muscles, and sagittal spinal alignment in young and middle-aged medical workers. *Clin. Biomechan. (Bristol, Avon)* 49 128–133. 10.1016/j.clinbiomech.2017.09.008 28934633

[B29] Michael J. W. Schlüter-Brust K. U. Eysel P. (2010). The epidemiology, etiology, diagnosis, and treatment of osteoarthritis of the knee. *Deutsches Arzteblatt Int.* 107 152–162. 10.3238/arztebl.2010.0152 20305774 PMC2841860

[B30] Mills K. Hunt M. A. Leigh R. Ferber R. (2013). A systematic review and meta-analysisof lower limb neuromuscular alterations associated with knee osteoarthritis during level walking. *Clin. Biomech.* 28 713–724. 10.1016/j.clinbiomech.2013.07.008 23953330

[B31] Ophir J. Cespedes I. Ponnekanti H. Yazdi Y. Li X. (1991). Elastography:a quantitative method for imaging the elasticity of biological tissues. *Ultrason. Imaging* 13 111–134. 10.1177/016173469101300201 1858217

[B32] Reginster J. (2002). The prevalence and burdenof arthritis. *Rheumatology* 41 3–6. 10.1093/rheumatology/41.S1.3 12173279

[B33] Rodrigues C. J. Rodrigues Junior A. J. (2000). A comparative study of aging of the elastic fiber system of the diaphragm and the rectus abdominis muscles in rats. *Braz. J. Med. Biol. Res.* 3 1449–1454. 10.1590/S0100-879X2000001200008 11105097

[B34] Sarvazyan A. P. Rudenko O. V. Swanson S. D. Fowlkes J. B. Emelianov S. Y. (1998). Shear wave elasticity imaging:a new ultrasonic technology of medical diagnostics. *Ultrasound Med. Biol.* 24 1419–1435. 10.1016/S0301-5629(98)00110-0 10385964

[B35] Sharma L. (2021). Osteoarthritis of the Knee. *N. Engl. J. Med.* 384 51–59. 10.1056/NEJMcp1903768 33406330

[B36] Sigrist R. M. S. Liau J. Kaffas A. E. Chammas M. C. Willmann J. K. (2017). Ultrasound elastography: review of techniques and clinical applications. *Theranostics* 7 1303–1329. 10.7150/thno.18650 28435467 PMC5399595

[B37] Simons D. G. (1996). Clinical and etiological update of myofascial pain from trigger points. *J. Musculoskel Pain* 4 93–122. 10.1300/J094v04n01_07

[B38] Taş S. Onur M. R. Yılmaz S. Soylu A. R. Korkusuz F. (2017). Shear wave elastography is a reliable and repeatable method for measuring the elastic modulus of the rectus femoris muscle and patellar tendon. *J. Ultrasound Med.* 36 565–570. 10.7863/ultra.16.03032 28108983

[B39] Zhou J. P. Yu J. F. Feng Y. N. Liu C. L. Su P. Shen S. H. (2020). Modulation in the elastic properties of gastrocnemius muscle heads in individuals with plantar fasciitis and its relationship with pain. *Sci. Rep.* 10:2770. 10.1038/s41598-020-59715-8 32066869 PMC7026110

[B40] Zhou J. Yu J. Liu C. Tang C. Zhang Z. (2019). Regional elastic properties of the achilles tendon is heterogeneously influenced by individual muscle of the gastrocnemius. *Appl. Bionics Biomech.* 2019:8452717. 10.1155/2019/8452717 31781292 PMC6874961

